# DeepARG: a deep learning approach for predicting antibiotic resistance genes from metagenomic data

**DOI:** 10.1186/s40168-018-0401-z

**Published:** 2018-02-01

**Authors:** Gustavo Arango-Argoty, Emily Garner, Amy Pruden, Lenwood S. Heath, Peter Vikesland, Liqing Zhang

**Affiliations:** 1 Department of Computer Science, Virginia Tech, Blacksburg, VA USA; 2 Department of Civil and Environmental Engineering, Virginia Tech, Blacksburg, VA USA

**Keywords:** Metagenomics, Antibiotic resistance, Deep learning, Machine learning

## Abstract

**Background:**

Growing concerns about increasing rates of antibiotic resistance call for expanded and comprehensive global monitoring. Advancing methods for monitoring of environmental media (e.g., wastewater, agricultural waste, food, and water) is especially needed for identifying potential resources of novel antibiotic resistance genes (ARGs), hot spots for gene exchange, and as pathways for the spread of ARGs and human exposure. Next-generation sequencing now enables direct access and profiling of the total metagenomic DNA pool, where ARGs are typically identified or predicted based on the “best hits” of sequence searches against existing databases. Unfortunately, this approach produces a high rate of false negatives. To address such limitations, we propose here a deep learning approach, taking into account a dissimilarity matrix created using all known categories of ARGs. Two deep learning models, DeepARG-SS and DeepARG-LS, were constructed for short read sequences and full gene length sequences, respectively.

**Results:**

Evaluation of the deep learning models over 30 antibiotic resistance categories demonstrates that the DeepARG models can predict ARGs with both high precision (> 0.97) and recall (> 0.90). The models displayed an advantage over the typical best hit approach, yielding consistently lower false negative rates and thus higher overall recall (> 0.9). As more data become available for under-represented ARG categories, the DeepARG models’ performance can be expected to be further enhanced due to the nature of the underlying neural networks. Our newly developed ARG database, DeepARG-DB, encompasses ARGs predicted with a high degree of confidence and extensive manual inspection, greatly expanding current ARG repositories.

**Conclusions:**

The deep learning models developed here offer more accurate antimicrobial resistance annotation relative to current bioinformatics practice. DeepARG does not require strict cutoffs, which enables identification of a much broader diversity of ARGs. The DeepARG models and database are available as a command line version and as a Web service at http://bench.cs.vt.edu/deeparg.

**Electronic supplementary material:**

The online version of this article (10.1186/s40168-018-0401-z) contains supplementary material, which is available to authorized users.

## Background

Antibiotic resistance is an urgent and growing global public health threat. It is estimated that the number of deaths due to antibiotic resistance will exceed ten million annually by 2050 and cost approximately 100 trillion USD worldwide [1–3]. Antibiotic resistance arises when bacteria are able to survive an exposure to antibiotics that would normally kill them or stop their growth. This process allows for the emergence of “superbugs” that are extremely difficult to treat. A few examples include methicillin-resistant *Staphylococcus aureus* (MRSA), which is an extremely drug-resistant bacterium associated with several infections [4], multidrug-resistant (MDR) *Mycobacterium tuberculosis*, which is resistant to rifampicin, fluoroquinolone, and isoniazid [5], and colistin-carbapenem-resistant *Escherichia coli*, which has gained resistance to last-resort drugs through the acquisition of the *mcr-1* and *bla*_*NDM-1*_ antibiotic resistance genes (ARGs) [6, 7].

The advent of high throughput DNA sequencing technology now provides a powerful tool to profile the full complement of DNA, including ARGs, derived from DNA extracts obtained from a wide range of environmental compartments. For example, ARGs have now been profiled using this kind of metagenomic approach in livestock manure, compost, wastewater treatment plants, soil, water, and other affected environments [8–13], as well as within the human microbiome [14, 15]. Identification of ARGs from such samples is presently based on the computational principle of comparison of the metagenomic DNA sequences against available online databases. Such comparison is performed by aligning raw reads or predicted open reading frames (full gene length sequences) from assembled contigs to the database of choice, using programs such as BLAST [16], Bowtie [17], or DIAMOND [18], and then predicting or assigning the categories of ARGs present using a sequence similarity cutoff and sometimes an alignment length requirement [19–21].

Existing bioinformatics tools focus on detecting known ARG sequences from within genomic or metagenomic sequence libraries and thus are biased towards specific ARGs [22]. For instance, ResFinder [20] and SEAR [23] predict specifically plasmid-borne ARGs, and Mykrobe predictor [24] is dedicated to 12 types of antimicrobials, while PATRIC [21] is limited to identifying ARGs encoding resistance to carbapenem, methicillin, and beta lactam antibiotics. Most of these tools use existing microbial resistance databases along with a “best hit” approach to predict whether a sequence is truly an ARG. Generally, predictions are restricted to high identity cutoffs, requiring a best hit with an identity greater than 80% by many programs such as ResFinder [20] and ARGs-OAP [8, 19, 20]. In some studies, the identity cutoff is even higher, as high as 90% for determining structure and diversity of ARGs through several resistomes [8] or analyzing the co-occurrence of environmental ARGs [25].

**Fig. 1:**
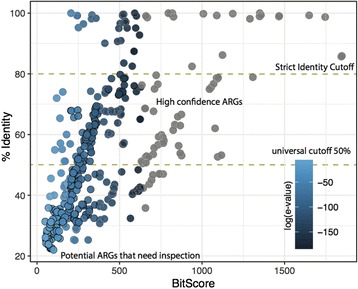
Bit score vs. identity distribution, illustrating the relationship between the UNIPROT genes against the CARD and ARDB genes in terms of the percentage identity, bit score, and e-value. Colors depict the exponent of the e-value (e-values below 1e-200 are represented by gray dots)

Although the best hit approach has a low false positive rate, that is, few non-ARGs are predicted as ARGs [9], the false negative rate can be very high and a large number of actual ARGs are predicted as non-ARGs [19, 22]. Figure 1 shows the distribution of manually curated potential ARGs from the Universal Protein Resource (UNIPROT) database [26] against the Comprehensive Antibiotic Resistance Database (CARD) [27] and the Antibiotic Resistance Genes Database (ARDB) [28]. All of the gene comparisons indicate significant e-values < 1e-20 with the sequence identity ranging from 20 to 60% and bit scores > 50, which is considered statistically significant [29]. Thus, high identity cutoffs clearly will remove a considerable number of genes that in reality are ARGs. For example, the entry O07550 (Yhel), a multidrug ARG conferring resistance to doxorubicin and mitoxantrone, has an identity of 32.47% with a significant e-value of 6e-77 to the best hit from the CARD database; the gene POCOZ1 (VraR), conferring resistance to vancomycin, has an identity of only 23.93% and an e-value 9e-13 to the best hit from the CARD database. Therefore, more moderate constraints on sequence similarity should be considered to avoid an unacceptable rate of false negatives. On the other hand, for short metagenomic sequences/reads (e.g., ~ 25aa or 100 bp), a stricter identity constraint of ~ 80% is recommended [20, 29] to avoid a high false positive rate. In principle, the best hit approach works well for detecting known and highly conserved categories of ARGs but may fail to detect novel ARGs or those with low sequence identity to known ARGs [19, 30].

To address the limitation of current best hit methodologies, a deep learning approach was used to predict ARGs, taking into account the similarity distribution of sequences in the ARG database, instead of only the best hit. Deep learning has proven to be the most powerful machine learning approach to date for many applications, including image processing [31], biomedical signaling [32], speech recognition [33], and genomic-related problems, such as the identification of transcription factor binding sites in humans [34, 35]. Particularly in the case of predicting DNA sequence affinities, the deep learning model surpasses all known binding site prediction approaches [34]. Here, we develop, train, and evaluate two deep learning models, DeepARG-SS and DeepARG-LS, to predict ARGs from short reads and full gene length sequences, respectively. The resulting database, DeepARG-DB, is manually curated and is populated with ARGs predicted with a high degree of confidence, greatly expanding the repertoire of ARGs currently accessible for metagenomic analysis of environmental datasets. DeepARG-DB can be queried either online or downloaded freely to benefit a wide community of users and to support future development of antibiotic resistance-related resources.

## Implementation

### Database merging

The initial collection of ARGs was obtained from three major databases: CARD [27], ARDB [28], and UNIPROT [26]. For UNIPROT, all genes that contained the Antibiotic Resistance keyword (KW-0046) were retrieved, together with their metadata descriptions when available. All identical or duplicate sequences were removed by clustering all the sequences (ARDB + CARD + UNIPROT) with CD-HIT [36], discarding all except one that had 100% identity and the same length. The remaining set of sequences comprised a total of 2290 genes from ARDB (50% of the original ARDB genes), 2161 from CARD (49% of the original CARD genes), and 28,108 from UNIPROT (70% of the original UNIPROT genes). This indicates a high redundancy of sequences within and among the ARG databases.

### ARG annotation of CARD and ARDB

The ARDB and CARD databases both contain information to aid in the classification of ARGs, including the antibiotic category to which a gene confers resistance (e.g., macrolides, beta lactamases, or aminoglycosides) and the antibiotic group to which the gene belongs (e.g., *tetA*, *sul1*, *macB*, *oxa*, *mir*, or *dha*). Manual inspection revealed that some genes have been assigned to specific sets of antibiotics instead of antibiotic resistance categories or categories. For instance, carbapenem, carbenicillin, cefoxitin, ceftazidime, ceftriaxone, and cephalosporin are actually a subset of the beta lactamases category. Thus, a total of 102 antibiotics that were found in the ARDB and CARD databases were further consolidated into 30 antibiotic categories (see Additional file 1: Table S1).

### UNIPROT gene annotation

Compared to the ARGs in CARD and ARDB, the UNIPROT genes with antibiotic resistance keywords are less well curated. Therefore, additional procedures were applied to further annotate the UNIPROT genes. Specifically, based on the CD-hit [36] clustering results, clusters that contained only UNIPROT genes were classified into two categories: 1) those without any annotation were tagged as “unknown” and 2) those with descriptions were text mined to identify possible association with antibiotic resistance.

**Fig. 2:**
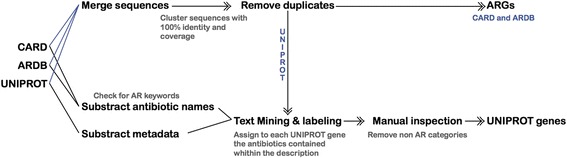
Preprocessing and UNIPROT ARGs annotation. Antibiotic resistance genes from CARD, ARDB, and UNIPROT were merged and clustered to remove duplicates. Then, sequences from UNIPROT are annotated using the matches between the metadata and the names of antibiotic categories from ARDB and CARD

UNIPROT’s sequence description contains a variety of features including a description of possible functions of the protein, the gene name based on HUGO nomenclature [37] for each sequence, and the evidence indicating whether a sequence has been manually inspected or not. A text mining approach was used to mine the genes’ descriptive features to identify their antibiotic resistance associations with the 30 antibiotic categories. The Levenshtein distance [38] was used to measure the similarities between gene description and antibiotic categories. This text mining approach was used because the names of the antibiotic resistance categories are not standardized among the databases and flexibility is needed to identify as many antibiotic associations as possible. For instance, genes linked to beta lactamases were sometimes tagged as beta-lactam, beta-lactamases, or beta-lactamase. Thus, text mining using all the alternative words allows comprehensive identification of antibiotic associations for each gene. Using this strategy, genes from UNIPROT were tagged either to their antibiotic resistance associations based on their description, or to “unknown” if no link to any antibiotic was found. Then, manual inspection was performed to remove misleading associations that passed the similarity criteria. The final set of genes and their tagged antibiotic resistance categories are shown in Fig. 2. Altogether, 16,360 UNIPROT genes remained after this refinement procedure.

**Fig. 3:**
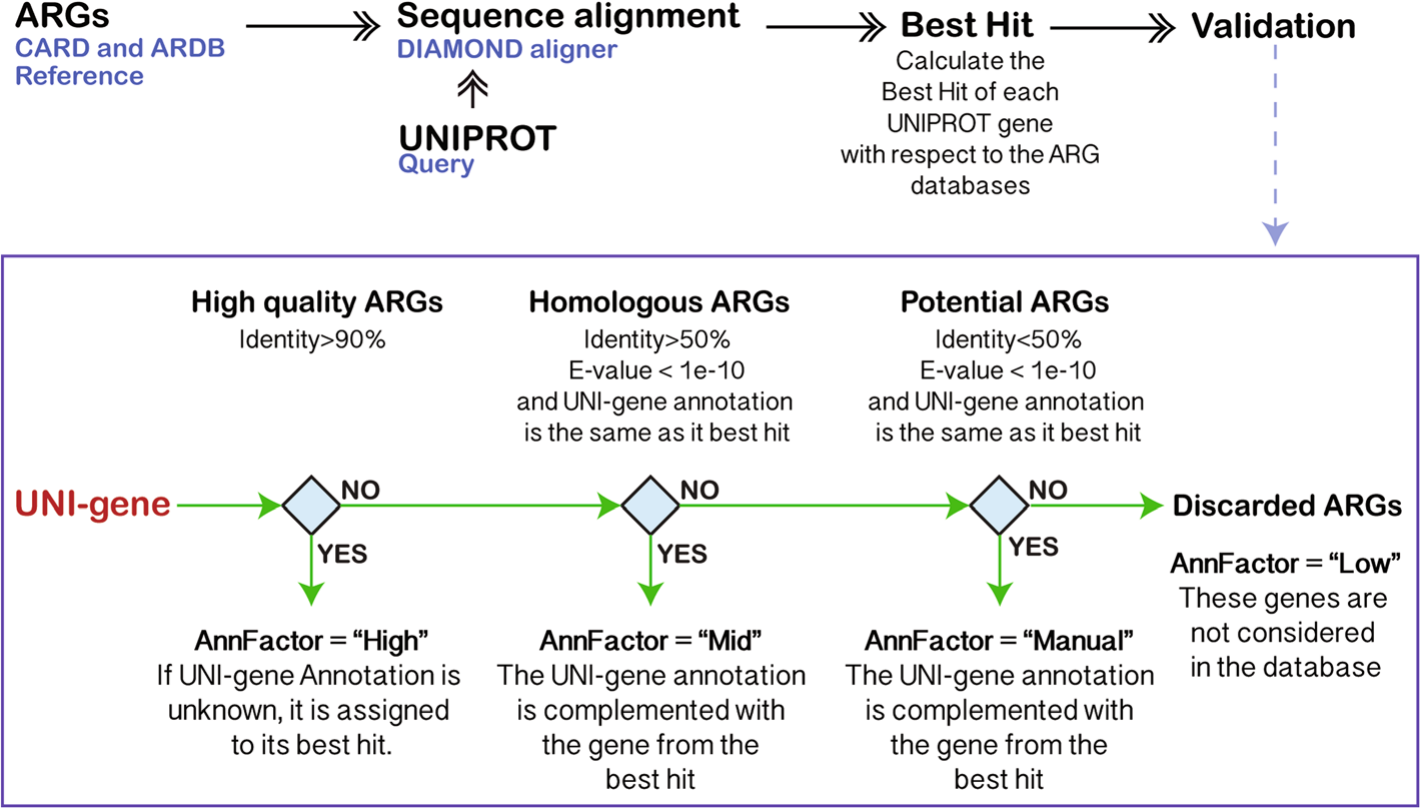
Validation of UNIPROT annotations. UNIPROT genes were aligned against the CARD and ARDB databases. The alignment with the highest bit score was selected for each UNI-gene (best hit) and a set of filters were applied to determine the UNI-gene annotation factor (AnnFactor)

The text mining procedure enabled the UNIPROT genes to become linked to one or more categories of antibiotics. However, the text mining procedure is purely based on gene metadata. Therefore, there was no evidence at the sequence level that the UNIPROT genes were truly associated with antibiotic resistance. For that reason, the UNIPROT gene’s annotation was further validated by their sequence identity to the CARD and ARDB databases. DIAMOND, a program that has similar performance to BLAST [39], but is much faster [18], was used for this purpose. For simplicity, UNI-gene is used here to denote a UNIPROT-derived gene, and CARD/ARDB-ARG is used to denote a gene derived from either CARD or ARDB (Fig. 3). According to the sequence identity, each UNI-gene was classified into the following categories based on their potential to confer antibiotic resistance defined as annotation factor:

1. High quality ARGs (High): A UNI-gene is tagged with a “High” annotation factor if it has ≥ 90% identity to a CARD/ARDB-ARG over its entire length. This similarity cutoff has been used in other studies to identify relevant ARGs [40, 41] and is stricter than that used in the construction of the ARDB database [28].
2. Homologous ARGs (Mid): A UNI-gene is tagged with a “Mid” annotation factor if it has ≥ 50 and ≤90% identity and an e-value lower than 1e-10 to a CARD/ARDB-ARG and also consistent annotation to the CARD/ARDB-ARG.
3. Potential ARGs (Manual Inspection): A UNI-gene is tagged with “Manual inspection” if it has < 50% identity and an e-value lower than 1e-10 to CARD/ARDB-ARGs and also consistent annotation to CARD/ARDB-ARGs. This gene is considered a potential ARG but with insufficient evidence and therefore warrants further analysis for the veracity of its antibiotic resistance.
4. Discarded ARGs (Low): A UNI-gene is discarded if its annotation differs from the best hit CARD/ARDB-ARG and the e-value is greater than 1e-10. Note that the gene can potentially still be an ARG, but due to a lack of sufficient evidence, it is removed from current consideration to ensure ARG annotation quality.

Altogether, 16,222 genes were tagged in the categories of “High” and “Mid” annotation factors. After removing sequences annotated as conferring resistance by single nucleotide polymorphisms (SNPs), a total of 10,602 UNIPROT, 2203 CARD, and 2128 ARDB genes were remaining for downstream analysis. In total, the DeepARG-DB comprises 14,933 genes including the three databases (CARD, ARDB, and UNIPROT). This database was used for the construction of the deep learning models.

### Deep learning

**Fig. 4:**
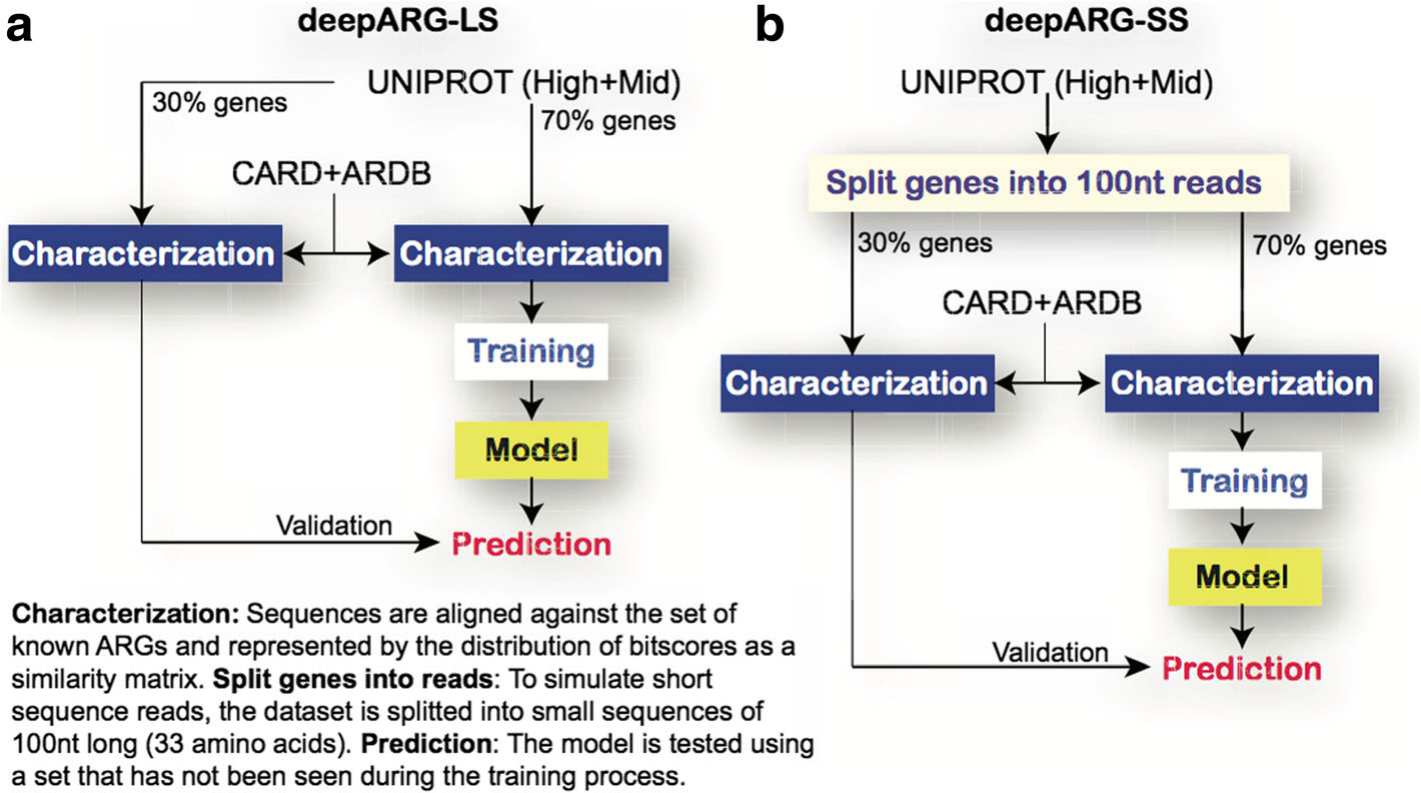
Classification framework. UNIPROT genes were used for validation and training whereas the CARD and ARDB databases were used as features. The distance between genes from UNIPROT to ARGs databases is computed using the sequence alignment bit score. Alignments are done using DIAMOND with permissive cutoffs allowing a high number of hits for each UNIPROT gene. This distribution is used to train and validate the deep learning models (The panel in the figure provides additional description on the training of the models)

Supervised machine learning models are usually divided into characterization, training, and prediction units. The characterization unit is responsible for the representation of DNA sequences as numerical values called features. It requires a set of DNA descriptors that are based on global or local sequence properties. Here, the concept of dissimilarity based classification [42] was used, where sequences were represented and featured by their identity distances to known ARGs. The CARD and ARDB genes were selected to represent known ARGs, whereas the UNIPROT (High+Mid) genes were used for training and validation of the models. DeepARG consists of two models: DeepARG-LS, which was developed to classify ARGs based on full gene length sequences, and DeepARG-SS, which was developed to identify and classify ARGs from short sequence reads (see Fig. 4). The bit score was used as the similarity indicator, because it takes into account the extent of identity between sequences and, unlike the e-value, is independent of the database size [29]. The process for computing the dissimilarity representation was carried out as follows. The UNIPROT genes were aligned to the CARD and ARDB databases [27, 28] using DIAMOND [18] with very permissive constraints: 10,000 maximum number of hits representing the total number of reported hits to which a UNIPROT gene is aligned, a 20% minimum identity (-id 20), and an e-value smaller than 1e-10. The bit score was then normalized to the [0, 1] interval to represent the sequence similarity as a distance. Hence, scores close to 0 represent small distance or high similarity, and scores around 1 represent distant alignments. Thus, a feature matrix was built where the rows correspond to the sequence similarity of the UNIPROT genes to the features (ARDB/CARD genes).

A deep learning model, DeepARG, was subsequently created to annotate metagenomic sequences to antibiotic resistance categories. One of the main advantages of deep learning over other machine learning techniques is its ability to discriminate relevant features without the need for human intervention [43–45]. It has been highlighted for its ability to resolve multiclass classification problems [34, 46–49]. Here, a deep learning multiclass model was trained by taking into account the identity distance distribution of a sequence to all known ARGs. This distribution represents a high level of sequence abstraction propagated through a fully connected network. The DeepARG model consists of four dense hidden layers of 2000, 1000, 500, and 100 units that propagate the bit score distribution to dense and abstract features. The input layer consists of 4333 units that correspond to the ARGs from ARDB and CARD. These features are used during training and evaluation. To avoid overfitting, random hidden units were removed from the model at different rates using the dropout technique [50]. Lastly, the output layer of the deep neural network consists of 30 units that correspond to the antibiotic resistance categories (see Additional file 1: Table S1). The output layer uses a softMax [51, 52] activation function that computes the probability of the input sequence against each ARG category. The probability is used to define the ARG category to which the input sequence belongs. The DeepARG architecture is implemented using the Python Lasagne [53] module, a high-level wrapper for the widely used Theano [54] deep learning library. Because deep learning demands intensive computational resources, the training was carried out using the GPU routines from Theano. However, heavy computation was required only once to obtain the deep learning model and the prediction routines do not require such computational resources.

Two strategies have generally been used to identify ARGs from metagenomic data; one predicts ARGs directly using short reads, while the other uses predicted open reading frames (i.e., full gene-length sequences) from assembled contigs to predict ARGs. To allow for both annotation strategies, two deep learning models, DeepARG-SS and DeepARG-LS, were developed to process short reads and full gene length sequences, respectively. The DeepARG-SS model was designed specially to classify short reads generated by NGS technologies such as Illumina. Therefore, ARGs are split into small sequences to simulate short sequence reads (see Fig. 4b). DeepARG-LS was trained using complete ARG sequences and can be used to annotate novel ARG genes (see Fig. 4a), for instance, in open reading frames detected in assembled contigs from the MetaHit consortium [55]. Note that each model was trained and validated separately to ensure high performance.

## Results and discussion

To evaluate the performance of the DeepARG models (DeepARG-SS and DeepARG-LS), five different experiments were conducted and compared to the best hit approach. The prediction quality was evaluated by precision, recall, and F1-score metrics defined as,

$$ Precision=\frac{TP}{TP+ FP}, $$

$$ Recall=\frac{TP}{TP+ FN}, $$

$$ F1\  score=2\ast \frac{precision\ast recall}{precision+ recall}, $$

where TP represents true positives (i.e., an ARG from the category of interest is predicted correctly as that ARG category), FP false positives (an ARG from a different category is predicted as from the category of interest), and FN false negatives (an ARG from the category of interest is predicted as a different ARG category).

Note because the first step of the DeepARG pipeline consists of the sequence alignment using DIAMOND, nonARGs (short reads or full length genes) are filtered out and not considered for further prediction. Therefore, the alignment stage only passes ARG-like sequences that have e-value < 1e-10 and identity > 20% to DeepARG for prediction. Thus, the performance reflects the capability of the DeepARG models in differentiating the 30 antibiotic resistance categories (see Additional file 1: Table S1).

### Antibiotic resistance database

**Fig. 5:**
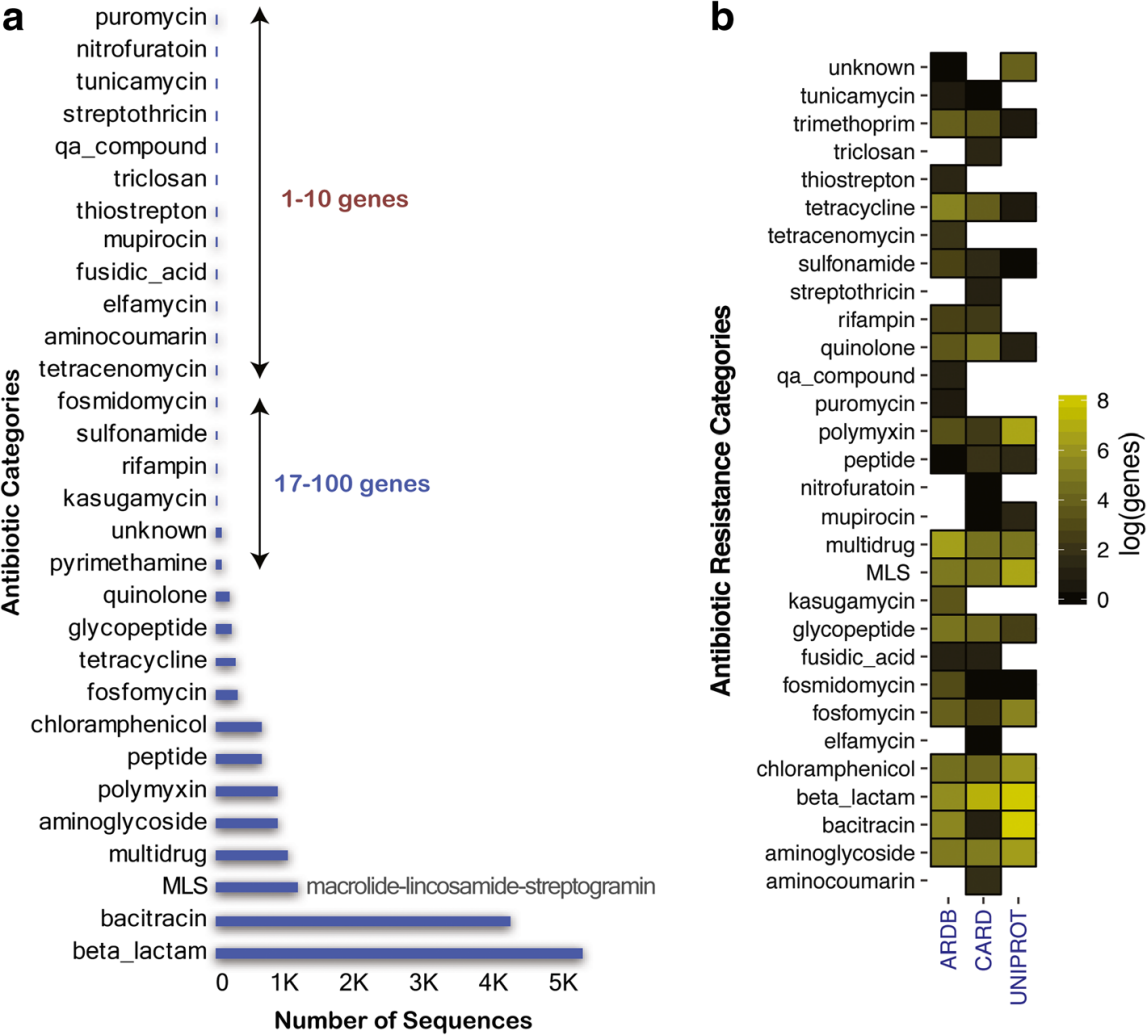
**a** Distribution of the number of sequences in the 30 antibiotic categories in DeepARG-DB. **b** The relative contribution of ARG categories in the ARDB, CARD, and UNIPROT databases

After the databases were merged and duplicates were removed, a total of 2161, 2290, and 28,108 genes were collected from the ARDB (50% of full ARDB), CARD (49% of all CARD genes), and UNIPROT (70% of total ARG-like sequences from UNIPROT) databases, respectively. For UNIPROT genes, a total of 16,360 genes were annotated using the available gene description. Following validation through sequence similarity and removing genes conferring resistance due to SNPs, 10,602 UNIPROT, 2203 CARD, and 2128 ARDB ARG sequences, remained. The resulting database, DeepARG-DB, comprises 30 antibiotic categories, 2149 groups, and 14,933 reference sequences (CARD+ARDB+UNIPROT). Over 34% of the genes belong to the beta lactamase category (5136), followed by 28% to the bacitracin category (4205), 7.4% to the macrolide-lincosamide-streptogramin (MLS) category (1109), 6.1% to the aminoglycoside category (915), 5.8% to the polymixin category (879), and 5.8% to the multidrug category (877, see Fig. 5a). The categories where the UNIPROT database made the greatest contribution correspond to beta-lactam, bacitracin, MLS, and polymyxin. However, not all ARG categories were found in the UNIPROT database, such as elfamycin, fusidic acid, and puromycin, among others (see Fig. 5b for details). One of the limitations of DeepARG-DB is its dependency on the quality of the CARD and ARDB databases. Thus, to avoid the propagation of errors from the CARD and ARDB, gene categories and groups were manually inspected and corrected, in particular, those annotations that differed between the ARDB and CARD databases. Because UNIPROT and CARD are continuously updated, the DeepARG-DB will likewise be updated and versioned accordingly as the trained deep learning models.

### Prediction of short sequence reads

**Fig. 6:**
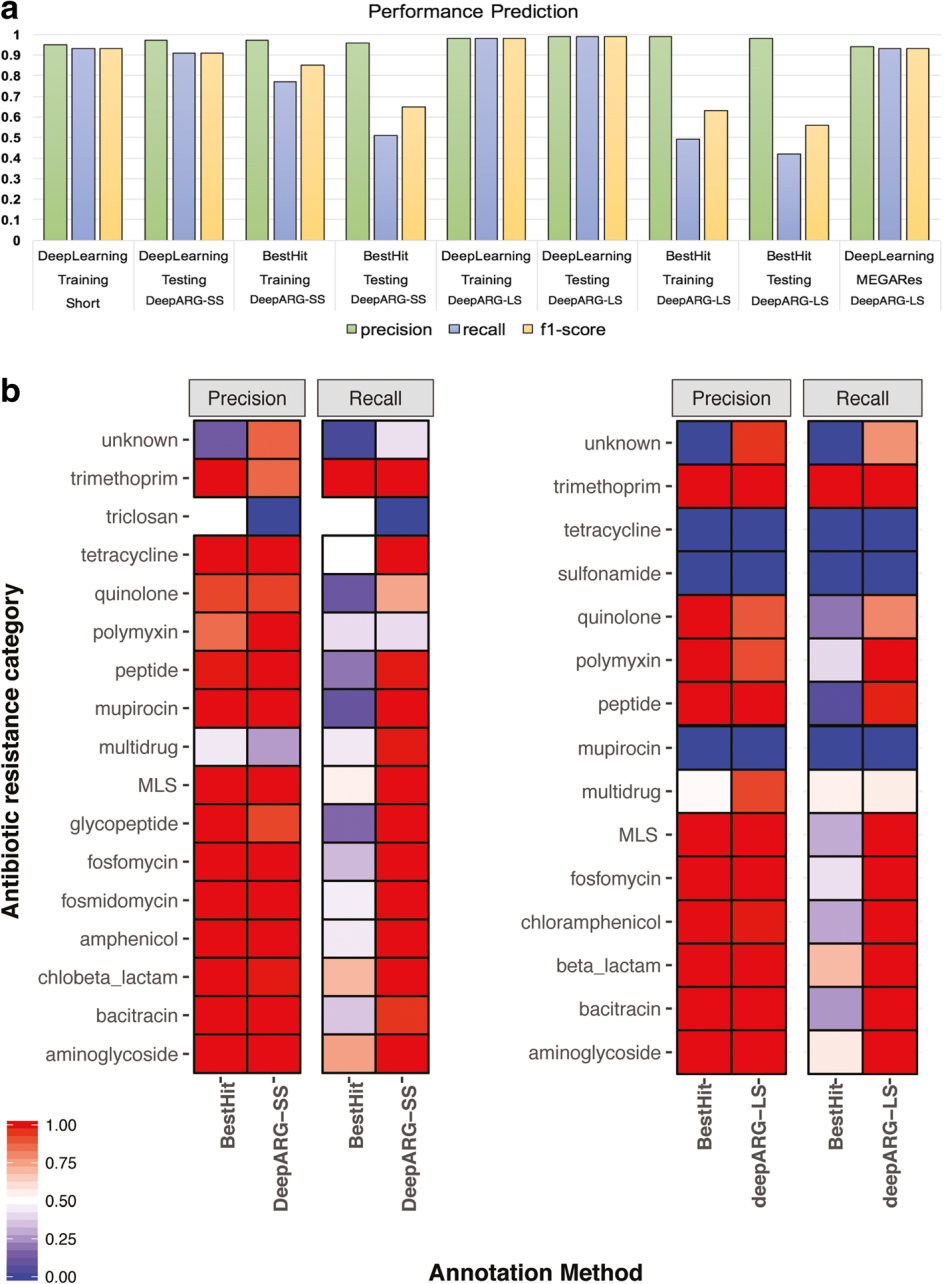
**a** Performance comparison of the DeepARG models with the best hit approach using precision, recall, and F1-score as metrics for the training and testing datasets. The MEGARes bars corresponds to the performance of DeepARG-LS using the genes from the MEGARes database. **b** Precision and recall of DeepARG models against the best hit approach for each individual category in the testing dataset. *UNIPROT genes are used for testing and not all the ARG categories have genes from the UNIPROT database

To simulate a typical metagenomic library, UNIPROT genes were split into 100 nucleotide long sequences, with a total of 321,008 reads generated. The DeepARG-SS model was subsequently trained and tested in a manner in which 70% of the reads were randomly selected for training, while the remaining 30% were reserved for validation. An overall precision of 0.97 and a recall of 0.91 were achieved among the 30 antibiotic categories tested (see Fig. 6a). In comparison, the best hit approach achieved an overall 0.96 precision and 0.51 recall. Achieving high precision for the best hit approach is not surprising, as the method relies on high identity constraints and has been reported to predict a low number of false positives, but a high number of false negatives [19]. We observed that both methods yielded high precision for most of the categories (see Fig. 6b). However, both methods performed poorly for the triclosan category, likely because the category was only represented by four genes in the database.

The DeepARG-SS model performed particularly well for antibiotic resistance categories that were well-populated, such as beta lactamases, bacitracin, and MLS, but not as well for categories represented by a small number of ARGs, such as triclosan and aminocoumarin. This result is expected due to the nature of neural network models. As more data becomes available to train the models, the better their ultimate performance. In contrast, the best hit approach yielded perfect prediction for some ARG categories containing a limited number of ARGs, but not for categories with a large number of ARGs (see Fig. 6b and Additional file 2: Table S2 for details).

For the multidrug antibiotic resistance category, the DeepARG-SS model had an almost perfect recall (0.99), implying that only a small number of multidrug reads were classified to other categories. However, the DeepARG-SS model also had the highest false positive rate compared to other categories (precision 0.27), implying that many non-multidrug reads were annotated as multidrug sequences. On the other hand, the best hit approach showed a higher precision (0.44), but a much lower recall (0.44). The multidrug category contains genes that confer resistance to multiple antibiotic categories such as macrolides, beta-lactamases, glycopeptides, quinolones, as well as other antimicrobials such as metals [56, 57]. These genes often share similar sequences, which makes it challenging for computational methods to determine the true identity of a short read. Therefore, when reads yield a best prediction probability less than 0.9, DeepARG reports the top two ARG categories for manual inspection. The low precision seen in both methods suggests that other non-multidrug categories may contain genes that have high sequence similarity to the multidrug category. This illustrates that there is still much room for improvement in existing databases.

Contrary to the multidrug category, the “unknown” antibiotic resistance category has a high precision of 0.87, but a low recall of 0.42, indicating a high false negative rate. Thus, reads from the unknown antibiotic resistance category can be mistakenly assigned/predicted as other antibiotic resistance categories. This highlights the need to check whether the unknown category actually contains genes from other ARG categories such as beta-lactam, macrolides, triclosan, among others. Comparatively, the best hit approach has the worst performance for the “unknown” antibiotic category (see Fig. 6b and Additional file 2: Table S2). In general, the DeepARG-SS model demonstrated significant improvement in the false negative rate compared to the best hit approach for nearly all ARG categories.

### Prediction of long ARG-like sequences

The DeepARG-LS model was trained and tested using full gene-length sequences. The UNIPROT validated genes were split into a training set (70% of the data) and a validation set (30% of the data) with the CARD and ARDB databases were used as features. The DeepARG-LS model shows similar results, with an overall precision of 0.99 and recall of 0.99 for predicting different categories of ARGs. Better performance in DeepARG-LS than DeepARG-SS is expected, because longer sequences contain more information than short reads (Fig. 6). Particularly DeepARG-LS achieved a high precision (0.97 ± 0.03) and an almost perfect recall (0.99 ± 0.01) for the antibiotic categories that were highly represented in the database, such as bacitracin, beta lactamase, chloramphenicol, and aminoglycoside (See Fig. 6b and Additional file 3: Table S3 for details). Comparatively, the best hit approach achieved a perfect precision (1.00 ± 0.00) but a much lower recall (0.48 ± 0.2) for these categories. Similar to DeepARG-SS, DeepARG-LS did not perform well for categories with few genes, such as sulfonamide and mupirocin (See Additional file 3: Table S3 for details).

### Performance prediction of known and validated ARGs

To further evaluate and validate performance, the DeepARG-LS model was applied to all of the ARG sequences in the MEGARes database [58]. This database contains manually curated ARGs from CARD [27], ARG-ANNOT [59], and RESFINDER [60]. ARGs conferring resistance by mechanisms that result from SNPs are removed in this test. Comparison of the DeepARG-LS prediction with the database annotation yielded an overall precision and recall of 0.94 and 0.93, respectively (Fig. 6 and Additional file 4: Table S4). The DeepARG-LS model achieved an almost perfect precision of 0.99 ± 0.05 and recall of 0.96 ± 0.03 for categories with a large number of genes, such as beta lactamases, elfamycin, fosfomycin, glycopeptides, MLS, and sulfonamide. However, the model performed poorly for categories that had a small number of genes (see Additional file 4: Table S4). For instance, MEGARes has a Tunicamycin gene that was assigned by the DeepARG-LS model as quinolone with a probability of 0.6. Such a low probability 0.6 suggests that the gene has more than one annotation. When the complete annotation for this gene was manually inspected, it was found that the DeepARG-LS model predicted the correct label (Tunicamycin) with a 0.3 probability, indicating that for this particular category more gene sequences are required to train the model. The DeepARG-DB database has only three Tunicamycin genes, which may explain why this gene was not properly classified. However, it is worth noting that the thiostrepton category was predicted correctly despite its lower number of training genes. The multidrug category is one of the most difficult categories to predict, containing about 200 genes. For the multidrug category, the DeepARG-LS model yielded a 0.7 precision with a 0.6 recall. This result suggests the need to manually inspect the genes tagged as multidrug as well as the genes from other categories that were assigned to the multidrug category. Challenges annotating genes belonging to the multidrug category further highlights the broader need to review, compare, and seek consensus among different antibiotic resistance databases.

### Validation through *Novel* ARGs

**Fig. 7:**
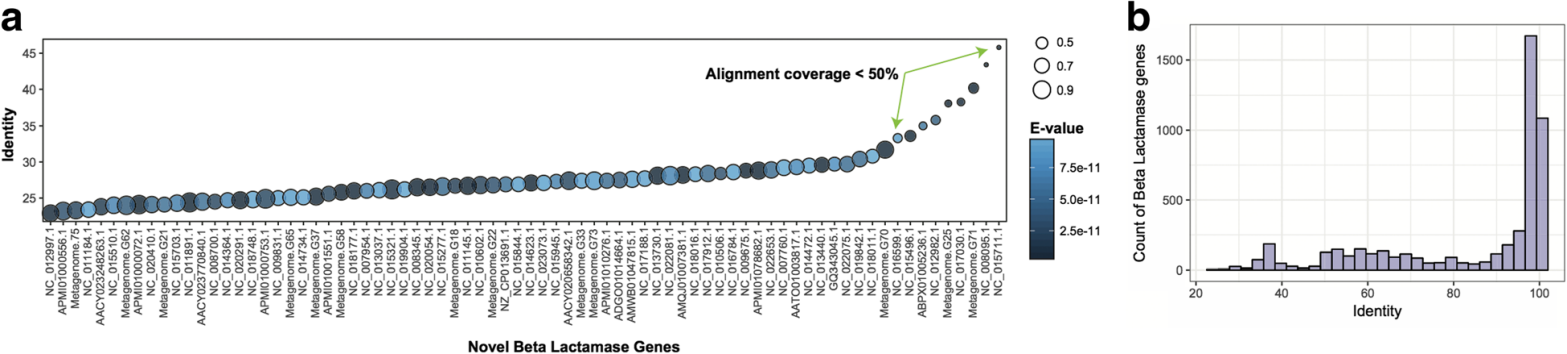
**a** Identity distribution of 76 novel beta lactamase genes against the DeepARG database (DeepARG-DB). Each dot corresponds to the best hit of each novel gene where color indicates the E-value (<1e-10) and size depicts the alignment coverage (> 40%). **b** Pairwise identity distribution of the beta lactamase genes in the DeepARG database

To test the ability of the DeepARG-LS model to predict novel ARGs, a set of 76 metallo beta lactamase genes were obtained from an independent study by Berglund et al. [61]. These novel genes have been experimentally validated via a functional metagenomics approach to confer resistance to carbapenem in *E. coli*. In the study, a large scale analysis was carried out by screening thousands of metagenomes and bacterial genomes to a curated set of beta lactamases. Using a hidden Markov model trained and optimized over a set of beta lactamases, 76 beta lactamase candidate novel genes were collected. Experimental validation was performed and 18 out of the 21 tested genes were able to hydrolase imipenem. Therefore, these 76 beta lactamase genes are expected to be mostly true ARGs and provide a unique opportunity to further test and validate the DeepARG-LS model. Interestingly, out of the 76 novel ARGs, the DeepARG-LS model was able to predict 65 (85% accuracy assuming all 76 are real ARGs) as the correct antibiotic category of beta lactamase with a probability greater than 0.99. The remaining nine genes were also predicted correctly by the DeepARG-LS model, but were filtered out because of their low alignment coverage (i.e., < 50%; alignment-length/ARG-length). Important to note is that the DeepARG-LS model was trained across 30 antibiotic categories and was not optimized to detect any one particular antibiotic category. Therefore, this result strongly demonstrates the capability of the DeepARG-LS model to detect novel ARGs. Of course, one possibility for the high accuracy of the DeepARG prediction is that these 76 genes and/or their closely related genes were included in training the DeepARG-LS model. To examine this possibility, the 76 beta lactamase genes were compared against all the sequences in DeepARG-DB using DIAMOND [18] and the best hit for each gene was extracted. Figure 7b shows that surprisingly, all of the best hits identified in DeepARG-DB had less than 40% sequence similarity to the 76 beta lactamases, indicating that the high accuracy of the DeepARG prediction is not due to inclusion of these genes and/or their close related genes in training the DeepARG-LS model. In fact, Fig. 7b shows the pairwise identity distribution of the beta lactamase genes used in training. Most of the beta lactamase genes are very similar to each other with pairwise identities greater than 90% and only a small number of them having low pairwise identity values. Taken together, these analyses show that using a diverse set of beta lactamase genes for training, the DeepARG-LS model, was able to learn the specificities of distantly related genes and consequently detect them. Thus, the DeepARG-LS model shows promise for the identification of novel ARGs. In contrast, the common practice of using the best hit approach with a universal 50% (or higher) identity cutoff [62] will fail to detect all these novel ARGs. Note that the length requirement imposed by DeepARG can be relaxed and adjusted depending on the specific research question. For example, if identifying as many potential novel ARGs as possible is the main focus, one can use a more relaxed length constraint than DeepARG’s default.

### Validation through an in Silico spike-in experiment

For metagenomic data sets derived from real-world samples, ARG reads may account for only a small fraction of the total reads. Thus, it is important to examine how the DeepARG-SS model performs in situations where non-target genes are dominant. In order to measure the ability of the DeepARG-SS model to discriminate/identify a small number of ARG reads among a large majority of nonARG reads, a negative metagenomic dataset was constructed that mimics a spike-in metagenomic experiment. First, a set of 6,485,966 reads of 100 bps were extracted from several eukaryote genomes (*Homo sapiens*, *Muss* muscle, and *Acanthisitta* cholirs) to generate the majority of nonARG reads (since eukaryote genomes are expected to have few ARG-like sequences). Second, a positive set of ARG reads was built by screening known ARGs against the bacterial genomes from the PATRIC database [63]. Only regions with an identity between 70 to 90% over the entire gene with an e-value below 1e-10 were used, and 10,000 short reads of 100 bps were extracted randomly from these regions to form the small set of ARG reads.

**Fig. 8:**
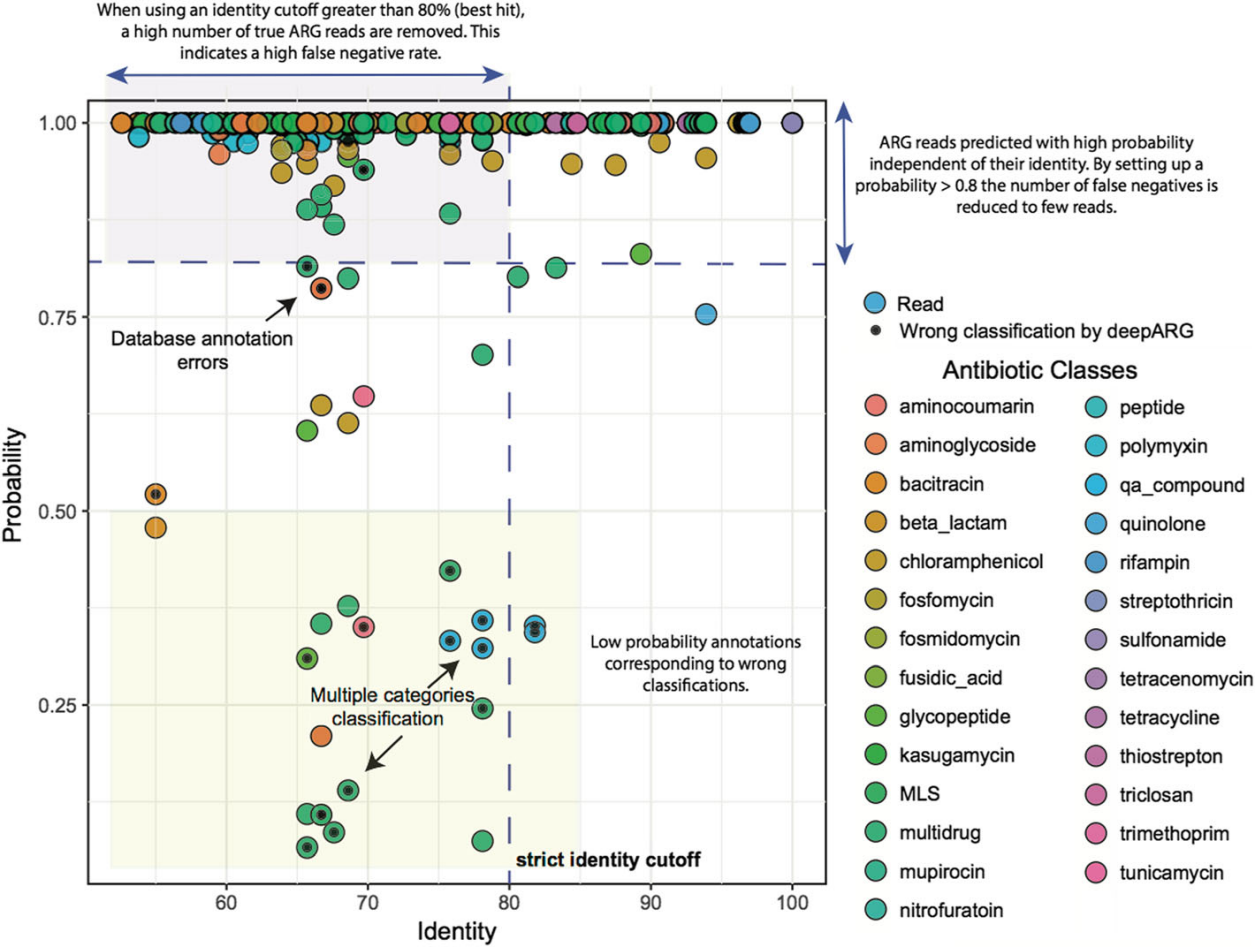
Prediction result using the DeepARG-SS model to classify ARGs for the spike-in dataset. Results for nonARG reads (eukaryotic reads) are not shown because DeepARG-SS was able to remove them during the alignment step using DIAMOND

Figure 8 shows the prediction result of DeepARG-SS for the 10,000 non-dominant ARG reads. Only one nonARG read was predicted to be a ARG read with an identity of 78%, while the remaining nonARG reads were discarded during the sequence alignment step due to failure to meet the requirement for a minimum of 20% sequence identity to at least one of the 4333 feature ARGs imposed by DeepARG. Thus, even though the dataset contains largely nonARG reads, the DeepARG-SS model was able to identify and predict the small number of ARG reads with high sensitivity. For example, using the default prediction probability cutoff of 0.8, the number of true positives (the ARG reads that were predicted to the correct antibiotic categories) is 9976, while the number of false negatives (the ARG reads that were predicted to the wrong antibiotic categories) was 24, yielding a 0.99 (9976/10000) sensitivity. These results show that, first, the alignment step in DeepARG acts as a filter that can effectively remove nonARG sequences, and second, despite the weak signal, DeepARG-SS predicts ARG reads correctly and with high sensitivity. Note that despite the ARG-like regions having 70–90% sequence identities to the known ARGs, the extracted reads have a much wider range of sequence identity of 50–100% to the ARGs due to different degrees of sequence conservation and diversity along the entire sequences of the ARGs (Fig. 8).

In practice, the annotation of short reads is often performed with the best hit approach. For this strategy, an identity cutoff between 80–90% to known ARGs is widely accepted as it has a low false positive rate [62]. When using the 80% cutoff, the best hit method yielded 4486 false negatives and 5514 true positives, thus a much lower sensitivity (0.55) than DeepARG. As expected, the best hit approach with these cutoffs can lead to underestimation or even erroneous inference of ARG contents in metagenomic datasets. Comparatively, the DeepARG-SS model aims to identify as many true positives as possible and, at the same time, to minimize the number of false negatives. To achieve this, the DeepARG-SS model examines the distribution of all the hits instead of relying on the best hit solely. As a result, the DeepARG-SS model was able to identify the correct antibiotic category and more importantly, to minimize the misclassification errors by providing a classification probability for each prediction. Our empirical analysis showed that this likelihood is an important metric to consider when one uses DeepARG for prediction. For instance, most of the classifications that have low prediction probabilities (< 0.5) are wrong and correspond to reads commonly found in different ARG categories, whereas only two erroneous predictions were observed for classification with high probabilities (> 0.8). Therefore, a probability cutoff of 0.8 is recommended when performing the classification. In addition, the DeepARG probability is independent of the sequence identity, which means that even with low sequence identities, the likelihood of obtaining the correct classification can still be high.

Still, it is important to clarify that despite the low false negative and false positive rate of this evaluation, the performance of the DeepARG models is dependent on the quality of the training database. As illustrated in Fig. 8, there are four incorrect classifications that have > 0.75 probability. These errors are likely generated by erroneous labels in the database. Hence, continued curation and/or validation of ARGs is crucial for improving the accuracy of ARGs predictions.

Also observed were several incorrect classifications with prediction probability < 0.5. The low probability for these reads suggests that they are predicted to multiple antibiotic categories. As a result, the probability is shared among different antibiotic categories. To avoid such errors, DeepARG uses a 0.8 minimum probability cutoff (as default) that can be modified by users. DeepARG also enables the adjustment of the identity cutoff used during the alignment stage. These parameters allow users to produce more or less stringent classification according to their needs.

### Validation through PseudoARGs

**Fig. 9:**
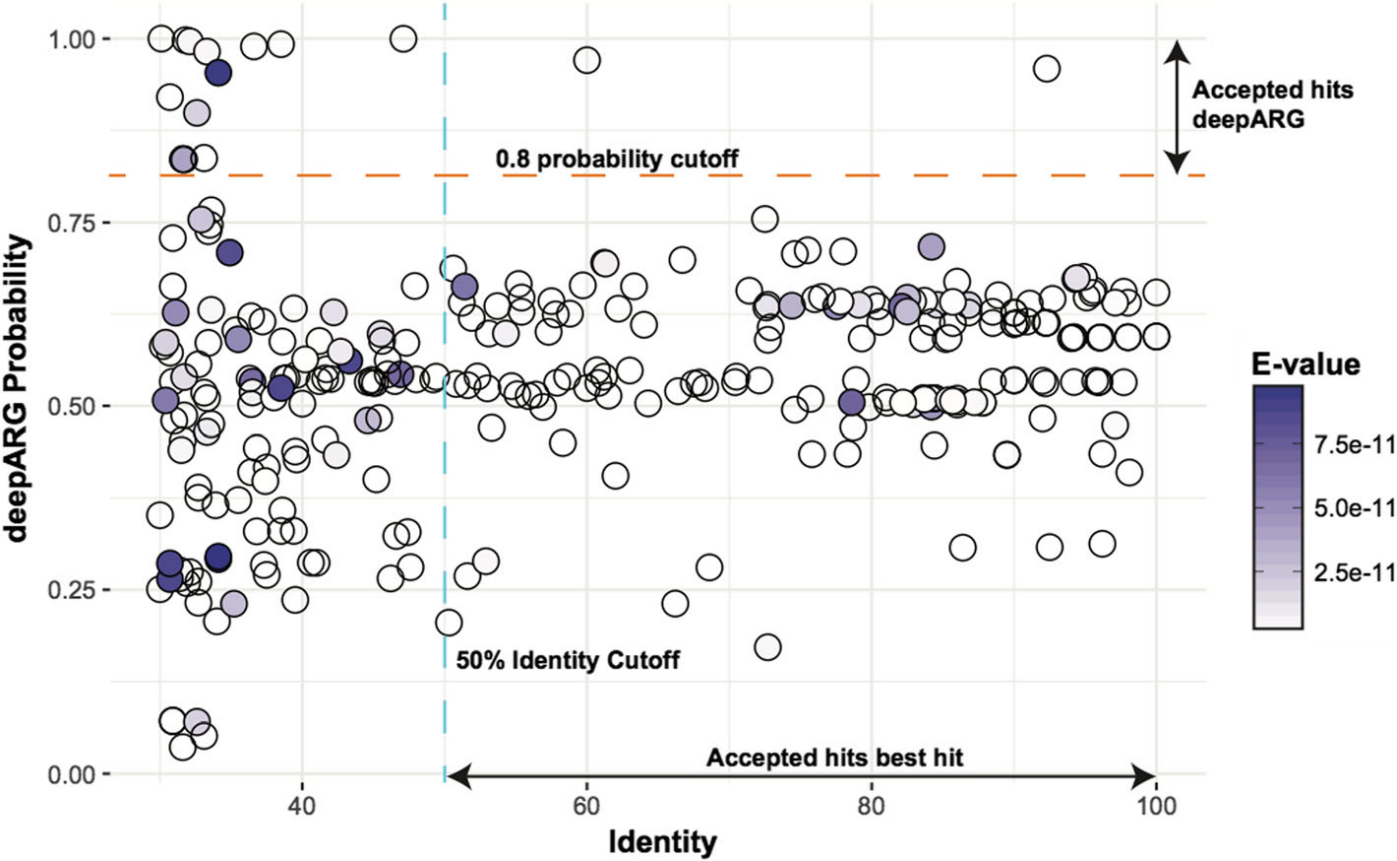
Distribution of DeepARG classification probability and the best hit identity. Each point indicates the alignment of each “partial” negative ARG against the DeepARG database. The horizontal line indicates the default setting for DeepARG predictions, i.e., the predictions with a probability higher than 0.8 are considered by DeepARG as high-quality classifications

To further examine the ability of DeepARG to discriminate genes that may contain segments of ARGs but are not true ARGs (i.e., pseudoARGs), a set of pseudoARGs were created. These genes were constructed by randomly picking k-mers from different ARG categories as follows: To build one gene, five k-mers of 50 amino acids long were randomly selected from one specific ARG category. Then, two 50-mers were randomly selected from ten more ARG categories. Finally, this process was repeated to build 300 genes with partial ARG content. This false positive dataset mimics the cases where genes from different categories share similarities within their sequences, e.g., the same domains or motifs. The pseudoARG dataset was then classified using the DeepARG-LS model and the best hit approach. As expected, the best hit approach was not able to filter out the false positive ARGs and produced a high false positive rate of 57% with the identity cutoff of 50% (Fig. 9), while using lower cutoffs would increase the number of false positives even more. In contrast, using the default classification probability cutoff of 0.8, the DeepARG-LS model was able to filter out 285 of the 300 pseudoARGs (5% false positive rate). This shows the superiority of the DeepARG-LS model in distinguishing pseudoARGs over the best hit approach, further supporting that the DeepARG model learns the uniqueness of the ARG categories through taking into account the similarities of the target sequence to all the ARG categories.

### Limitation of DeepARG and usage recommendation

The two DeepARG models, DeepARG-LS and DeepARG-SS, are tailored to different ARG prediction strategies. For example, it is now a common practice for researchers to collect different environmental samples, sequence the DNA to obtain metagenomic data, and use the data to address the question “what kinds of ARGs are present in the samples?”. In this case, with the metagenomic data, one can simply predict which ARG categories the reads belong to by applying the DeepARG-SS model directly to the reads, similar to what was done for the in silico spike-in metagenomic experiment. This task can be done rapidly as experiments demonstrated that predicting 100 million short reads required only 50 min on a personal MacBook pro with i7 processor and 16Gb of ram. As pointed out previously, training the DeepARG model is very time consuming, but is only done once. Alternatively, one can first assemble the short reads into contigs, obtain open reading frames (ORFs) using an ORF identification/prediction program for the contigs, and then run the DeepARG-LS model on the ORFs to predict ARG categories. Comparatively, the latter strategy can be much slower as it involves sequence assembly, but the prediction might be more accurate than direct prediction on reads. This is expected as the longer the sequences are, the more information contained, and therefore the more confidence one has for ARG prediction. This is also clear from the results where the DeepARG-LS model performed better than the DeepARG-SS model (Fig. 6). In cases where full gene length sequences are readily obtainable such as the 76 novel beta lactamase genes, DeepARG-LS can be deployed to predict the corresponding ARG categories.

Several points are worthy of discussion. First, the DeepARG models were trained across 30 ARG categories and are intended to predict which of these categories a gene or short read belongs to. It is not intended and cannot be used to predict antibiotic resistance that arises from SNPs. Second, the DeepARG models can only predict whether a gene or read belongs to one of the 30 categories that are considered by the model. If the gene or read belongs to an entirely new ARG category, DeepARG will not be able to predict it. In such a case, it is worth noting that prediction probabilities for the 30 categories are expectedly low and one should treat the predictions with caution and may discard the prediction if a high-quality set of ARG prediction is desired. Third, the performance of the DeepARG models hinges on the quality of the training database; i.e., the higher quality the training data, the higher prediction accuracy the model. Detailed analyses of the prediction results suggest that some of the ARG categories may have annotation errors, especially the multidrug and “unknown” categories, which in turn adversely affects the prediction of the models. This highlights the importance of continued and synergistic effort from the research community in curating and improving ARG nomenclature and annotation databases. Fourth, as with *all* in silico prediction, the DeepARG models can be used to get an overview or inference of the kinds of antibiotic resistance in a collection of sequences; strictly speaking, downstream experimental validation is required to confirm whether the sequences truly confer resistance.

## Conclusions

Here, a new computational resource for the identification and annotation of ARGs derived from metagenomic data is developed, trained, and evaluated. The deep learning approach proved to be more accurate than the widely used best hit approach and is not restricted to strict cutoffs, thus greatly reducing false negatives and offering a powerful approach for metagenomic profiling of ARGs in environmental compartments. Further, the DeepARG database developed here greatly expands the available ARGs individually available in the currently most widely used CARD, ARDB, and UNIPROT databases, including their existing sequence content and extensive metadata. DeepARG provides a publicly-available database structured into a simple category and group hierarchy for each ARG. While DeepARG is not intended to replace CARD or ARDB, in conjunction with deep learning, it aims to improve the ARG annotation by drastically reducing the false negative rate, while maintaining a similarly high true positive rate associated with the traditional best hit approach. The performance of DeepARG highly depends on the quality of the training database. Therefore, the inclusion of new entries based on the alignment’s similarity could integrate genes that have not been validated to produce antibiotic resistance in vivo. However, this in silico gene’s integration is useful to expand the diversity of ARGs, as it is shown by the analysis of novel ARGs where distant genes have been predicted to the correct antibiotic resistance category.

## Availability and requirements

DeepARG consists of a command line program where the input can be either a FASTA file or a BLAST tabular file. If the input is a FASTA sequence file, DeepARG will perform the sequence search first and then annotate ARGs. If the input is already a BLAST tabular file, DeepARG will annotate ARGs directly. An online version of DeepARG is also available where a user can upload a metagenomics raw sequence files (FASTQ format) for ARG annotation (http://bench.cs.vt.edu/deeparg). Once the data is processed, the user receives an email with results of annotated ARGs with the absolute abundance of the ARGs and the relative abundance of ARGs normalized to the 16S rRNA content in the sample as used in [19, 64]. This normalization is useful to compare the ARG content from different samples. The web service also allows users to modify the parameters (identity, probability, coverage, and E-value) of the DeepARG analysis. With the command line version, the user also has access to more elaborated results such as the probabilities of each read/gene belonging to the specific antibiotic resistance categories. In addition to prediction of antibiotic categories and the associated probabilities, the DeepARG model reports the entries with multiple classifications. In detail, if a read or complete gene sequence is classified to an antibiotic category with a probability below 0.9, the top two classifications will be provided. This would help researchers identify reads/sequences with less confident predictions, and it is recommended that the detailed output be examined together with domain knowledge to determine the more likely ARG category. The DeepARG-DB is freely available under the DeepARG Web site (http://bench.cs.vt.edu/deeparg) as a protein FASTA file and it is included into the git repository. Each entry in the database has a complete description that includes the gene identifier, the database where the gene is coming from, the antibiotic category, and the antibiotic group. For users interested on a particular set of genes, DeepARG also provides the steps to create a new deep learning model using the architecture of DeepARG. This architecture is not restricted to ARGs and can be used to train any set of genes.

## Additional files

Additional file 1: Table S1. Detected antibiotic names from the CARD and ARDB databases. Each antibiotic is grouped by the class of antibiotics. (PDF 65 kb)

Additional file 2: Table S2. Prediction performance of the individual ARG categories for the deepARG-SS model and the Best Hit approach using the UNIPROT 70% genes for training and 30% for validation, where, genes are split into 100 nt long kmers to simulate next generation short sequences. (PDF 73 kb)

Additional file 3: Table S3. Prediction performance of the individual ARG categories for the deepARG-LS model and the Best Hit approach using the UNIPROT 70% genes for training and 30% for validation. (PDF 71 kb)

Additional file 4: table S4. Prediction results of the genes from MEGARes using the deepARG-LS model trained with our database. (PDF 60 kb)

## Abbreviations

ARG: Antibiotic resistance gene
FN: False negatives
FP: False positives
TP: True positives
