# Supplementary material for: DeepARG: a deep learning approach for predicting antibiotic resistance genes from metagenomic data
Source: Microbiome. 2018 Feb 1;6:23. doi: 10.1186/s40168-018-0401-z (PMC5796597; doi:10.1186/s40168-018-0401-z)
Supplement: Supplementary file 1 — Detected antibiotic names from the CARD and ARDB databases. Each antibiotic is grouped by the class of antibiotics. (PDF 65 kb) [file 40168_2018_401_MOESM1_ESM.pdf]

**Antibiotic**	**Antibiotic class**
6_n_netilmicin	aminoglycoside
acriflavin	acriflavin
acriflavine	acriflavine
amikacin	aminoglycoside
aminocoumarin	aminocoumarin
aminoglycoside	aminoglycoside
apramycin	aminoglycoside
astromicin	aminoglycoside
bacitracin	bacitracin
beta_lactam	beta_lactam
bleomycin	bleomycin
butirosin	aminoglycoside
carbapenem	beta_lactam
carbenicillin	beta_lactam
cefoxitin	beta_lactam
ceftazidime	beta_lactam
ceftriaxone	beta_lactam
cephalosporin	beta_lactam
cephalosporin_i	beta_lactam
cephalosporin_ii	beta_lactam
cephalosporin_iii	beta_lactam
cephamycin	beta_lactam
chloramphenicol	chloramphenicol
ciprofloxacin	quinolone
cloxacillin	beta_lactam
deoxycholate	deoxycholate
dibekacin	aminoglycoside
doxorubicin	doxorubicin
e_cephalosporin	beta_lactam
e_penicillin	beta_lactam
efflux	multidrug

elfamycin	elfamycin
enoxacin	quinolone
erythromycin	macrolide-lincosamide-streptogramin
ethambutol	ethambutol
fluoramphenicol	fluoramphenicol
fluoroquinolone	quinolone
fosfomycin	fosfomycin
fosmidomycin	fosmidomycin
fusaric_acid	fusaric_acid
fusidic acid	fusidic_acid
fusidic_acid	fusidic_acid
gentamicin	aminoglycoside
gentamicin_b	aminoglycoside
gentamincin_b	aminoglycoside
glycopeptide	glycopeptide
glycylcycline	tetracycline
hygromycin_b	aminoglycoside
isepamicin	aminoglycoside
isoniazid	isoniazid
kanamycin	aminoglycoside
kasugamycin	kasugamycin
lincomycin	macrolide-lincosamide-streptogramin
lincosamide	macrolide-lincosamide-streptogramin
linezolid	linezolid
lipopeptide	lipopeptide
lividomycin	aminoglycoside
macrolide	macrolide-lincosamide-streptogramin
methicillin	beta_lactam
monobactam	beta_lactam
multidrug	multidrug
mupirocin	mupirocin
n_cephalosporin	beta_lactam

na_antimicrobials	multidrug
neomycin	aminoglycoside
netilmicin	aminoglycoside
nitrofuratoin	nitrofuratoin
norfloxacin	quinolone
paromomycin	aminoglycoside
penicillin	beta_lactam
peptide	peptide
phenicol	phenicol
pleuromutilin	pleuromutilin
polyamine	polyamine
polymyxin	polymyxin
puromycin	puromycin
pyrazinamide	pyrazinamide
qa_compound	qa_compound
ribostamycin	aminoglycoside
rifampin	rifampin
roxithromycin	roxithromycin
sisomicin	aminoglycoside
spectinomycin	aminoglycoside
spectomycin	aminoglycoside
streptogramin	macrolide-lincosamide-streptogramin
streptogramin_a	macrolide-lincosamide-streptogramin
streptogramin_b	macrolide-lincosamide-streptogramin
streptomycin	aminoglycoside
streptothricin	streptothricin
sulfonamide	sulfonamide
t_chloride	t_chloride
teicoplanin	glycopeptide
tetracenomycin_c	tetracenomycin
tetracycline	tetracycline
thiostrepton	thiostrepton

tigecycline	tetracycline
tobramycin	aminoglycoside
triclosan	triclosan
trimethoprim	trimethoprim
tunicamycin	tunicamycin
vancomycin	glycopeptide
viomycin	viomycin
novobiocin	aminocoumarin

Table S1: Detected antibiotic names from the CARD and ARDB databases. Each antibiotic is grouped by the class of antibiotics.
