# Supplementary material for: DeepARG: a deep learning approach for predicting antibiotic resistance genes from metagenomic data
Source: Microbiome. 2018 Feb 1;6:23. doi: 10.1186/s40168-018-0401-z (PMC5796597; doi:10.1186/s40168-018-0401-z)
Supplement: Supplementary file 2 — Prediction performance of the individual ARG categories for the deepARG-SS model and the Best Hit approach using the UNIPROT 70% genes for training and 30% for validation, where, genes are split into 100 nt long kmers to simulate next generation short sequences. (PDF 73 kb) [file 40168_2018_401_MOESM2_ESM.pdf]

Deep Learning Training performance

	**precision**	recall	f1-score
aminocoumarin	0.99	1	0.99
aminoglycoside	1	0.99	0.99
bacitracin	1	0.96	0.98
beta_lactam	0.99	0.99	0.99
chloramphenicol	1	0.97	0.99
elfamycin	1	1	1
fosfomycin	1	1	1
fosmidomycin	0.68	1	0.81
fusidic_acid	0.9	1	0.95
glycopeptide	0.99	1	1
kasugamycin	1	1	1
macrolide-lincosamide-streptogramin	1	1	1
multidrug	0.65	0.99	0.79
mupirocin	1	1	1
nitrofuratoin	1	1	1
peptide	1	0.98	0.99
polymyxin	1	0.41	0.58
puromycin	1	1	1
trimethoprim	1	1	1
qa_compound	1	1	1
quinolone	1	0.92	0.96
rifampin	1	1	1
streptothricin	1	1	1
sulfonamide	1	1	1
tetracenomycin	1	1	1
tetracycline	1	0.99	0.99
thiostrepton	1	1	1
triclosan	0.99	1	1
tunicamycin	1	1	1
unclassified	0	0	0
unknown	0.89	0.43	0.58
avg/total	0.95	0.93	0.93

Performance of the Deep Learning with the test dataset

	**precision**	**recall**	**f1-score**
aminocoumarin	0	0	0
aminoglycoside	1	1	1
bacitracin	1	0.95	0.98
beta_lactam	0.99	1	1
chloramphenicol	1	1	1
fosfomycin	1	1	1
fosmidomycin	1	1	1
glycopeptide	0.92	1	0.96

macrolide-lincosamide-streptogramin	1	1	1
multidrug	0.27	0.99	0.43
mupirocin	1	1	1
peptide	1	0.99	0.99
polymyxin	1	0.41	0.58
trimethoprim	0.86	1	0.92
quinolone	0.93	0.74	0.82
tetracycline	1	1	1
triclosan	0	0	0
unknown	0.87	0.42	0.57
avg/total	0.97	0.91	0.91

Best Hit training performance

	**precision**	**recall**	**f1-score**
aminocoumarin	0.98	1	0.99
aminoglycoside	1	0.89	0.94
bacitracin	1	0.62	0.76
beta_lactam	1	0.86	0.92
chloramphenicol	1	0.7	0.82
elfamycin	1	1	1
fosfomycin	1	0.79	0.88
fosmidomycin	0.78	0.96	0.86
fusidic_acid	0.89	0.86	0.88
glycopeptide	1	0.99	0.99
kasugamycin	1	1	1
macrolide-lincosamide-streptogramin	1	0.74	0.85
multidrug	0.87	0.9	0.88
mupirocin	1	0.77	0.87
nitrofuratoin	1	1	1
peptide	0.98	0.3	0.46
polymyxin	0.88	0.63	0.73
puromycin	1	1	1
trimethoprim	1	1	1
qa_compound	1	1	1
quinolone	0.98	0.88	0.93
rifampin	1	1	1
streptothricin	1	1	1
sulfonamide	1	1	1
tetracenomycin	1	1	1
tetracycline	1	0.99	0.99
thiostrepton	1	1	1
triclosan	1	1	1
tunicamycin	1	1	1
unknown	0.3	0.11	0.16
avg/total	0.97	0.77	0.85

Performance of Best Hit with the test dataset			
		80% identity	
	**precision**	recall	f1-score
aminoglycoside	1	0.75	0.86
bacitracin	1	0.36	0.53
beta_lactam	1	0.7	0.82
chloramphenicol	1	0.44	0.62
fosfomycin	1	0.45	0.62
fosmidomycin	1	0.33	0.5
glycopeptide	1	0.17	0.29
macrolide-lincosamide-streptogramin	1	0.55	0.71
multidrug	0.44	0.44	0.44
mupirocin	1	0.1	0.18
peptide	0.99	0.2	0.33
polymyxin	0.85	0.41	0.56
trimethoprim	1	1	1
quinolone	0.92	0.11	0.2
tetracycline	1	0.5	0.67
unknown	0.13	0.02	0.04
avg/total	0.96	0.51	0.65

Table S2: Prediction pefromance of the indiviual ARG categories for the deepARG-SS model and the Best Hit approach using the UNIPROT 70% genes for training and 30% for validation, where, genes are splitted into 100nt long kmers to simulate next generation short sequences.
