# Supplementary material for: DeepARG: a deep learning approach for predicting antibiotic resistance genes from metagenomic data
Source: Microbiome. 2018 Feb 1;6:23. doi: 10.1186/s40168-018-0401-z (PMC5796597; doi:10.1186/s40168-018-0401-z)
Supplement: Supplementary file 3 — Prediction performance of the individual ARG categories for the deepARG-LS model and the Best Hit approach using the UNIPROT 70% genes for training and 30% for validation. (PDF 71 kb) [file 40168_2018_401_MOESM3_ESM.pdf]

Deep learning training performance			
	precision	recall	f1-score
aminocoumarin	0.8	0.57	0.67
aminoglycoside	0.96	0.99	0.98
bacitracin	0.99	1	1
beta_lactam	1	1	1
chloramphenicol	1	1	1
elfamycin	1	0.6	0.75
fosfomycin	1	1	1
fosmidomycin	0.71	1	0.83
fusidic_acid	1	0.6	0.75
glycopeptide	0.99	0.96	0.98
kasugamycin	1	1	1
macrolide-lincosamide-streptogramin	1	0.99	0.99
multidrug	0.95	0.85	0.9
mupirocin	0.2	0.75	0.32
nitrofuratoin	0	0	0
peptide	0.99	0.98	0.99
polymyxin	0.89	0.99	0.94
puromycin	0	0	0
trimethoprim	1	0.99	0.99
qa_compound	1	1	1
quinolone	0.98	0.89	0.93
rifampin	1	1	1
streptothricin	1	0.33	0.5
sulfonamide	1	1	1
tetracenomycin	1	1	1
tetracycline	0.99	0.97	0.98
thiostrepton	0.8	1	0.89
triclosan	1	0.75	0.86
tunicamycin	0	0	0
unknown	0.93	0.86	0.89
avg/total	0.98	0.98	0.98

Performance of the Deep Learning with the test dataset			
	precision	recall	f1-score
aminoglycoside	1	1	1
bacitracin	1	1	1
beta_lactam	1	1	1
chloramphenicol	0.99	1	1
fosfomycin	1	1	1
macrolide-lincosamide-streptogramin	1	1	1
multidrug	0.92	0.56	0.7

mupirocin	0	0	0
peptide	1	0.98	0.99
polymyxin	0.91	1	0.95
trimethoprim	1	1	1
quinolone	0.89	0.8	0.84
sulfonamide	0	0	0
tetracycline	0	0	0
unclassified	0	0	0
unknown	0.95	0.78	0.86
avg/total	0.99	0.99	0.99

Best Hit training performance			
	precision	recall	f1-score
aminocoumarin	1	1	1
aminoglycoside	1	0.51	0.67
bacitracin	1	0.31	0.47
beta_lactam	1	0.59	0.74
chloramphenicol	1	0.42	0.59
elfamycin	1	1	1
fosfomycin	1	0.58	0.73
fosmidomycin	1	0.94	0.97
fusidic_acid	0.67	0.4	0.5
glycopeptide	1	0.83	0.91
kasugamycin	1	1	1
macrolide-lincosamide-streptogramin	1	0.44	0.61
multidrug	0.89	0.88	0.88
mupirocin	1	0.25	0.4
nitrofuratoin	1	1	1
peptide	0.97	0.06	0.11
polymyxin	0.99	0.41	0.58
puromycin	1	1	1
trimethoprim	1	0.99	0.99
qa_compound	1	1	1
quinolone	0.99	0.88	0.93
rifampin	1	1	1
streptothricin	1	1	1
sulfonamide	1	0.95	0.98
tetracenomycin	1	1	1
tetracycline	1	0.9	0.95
thiostrepton	1	1	1
triclosan	1	1	1
tunicamycin	1	1	1
unknown	0.75	0.08	0.14
avg/total	0.99	0.49	0.63

Performance of Best Hit with the test dataset			
	precision	recall	f1-score
aminoglycoside	1	0.57	0.73
bacitracin	1	0.26	0.42
beta_lactam	1	0.69	0.81
chloramphenicol	1	0.29	0.45
fosfomycin	1	0.42	0.6
macrolide-lincosamide-streptogramin	1	0.3	0.46
multidrug	0.52	0.55	0.53
mupirocin	0	0	0
peptide	1	0.06	0.12
polymyxin	1	0.4	0.57
trimethoprim	1	1	1
quinolone	1	0.2	0.33
sulfonamide	0	0	0
tetracycline	0	0	0
unknown	0	0	0
avg/total	0.98	0.42	0.56

Table S3: Prediction pefromance of the indiviual ARG categories for the deepARG-LS model and the Best Hit approach using the UNIPROT 70% genes for training and 30% for validation
