# Supplementary material for: DeepARG: a deep learning approach for predicting antibiotic resistance genes from metagenomic data
Source: Microbiome. 2018 Feb 1;6:23. doi: 10.1186/s40168-018-0401-z (PMC5796597; doi:10.1186/s40168-018-0401-z)
Supplement: Supplementary file 4 — Prediction results of the genes from MEGARes using the deepARG-LS model trained with our database. (PDF 60 kb) [file 40168_2018_401_MOESM4_ESM.pdf]

| | precision | recall | f1-score |
|---|---|---|---|
| /lycobacterium tuberculosis-specific Drug | 0 | 0 | 0 |
| Trimethoprim | 1 | 0.95 | 0.98 |
| Tunicamycin | 0 | 0 | 0 |
| aminocoumarin | 0.73 | 0.5 | 0.59 |
| aminoglycoside | 0.96 | 0.96 | 0.96 |
| bacitracin | 1 | 0.5 | 0.67 |
| beta_lactam | 1 | 0.99 | 1 |
| chloramphenicol | 0.87 | 0.82 | 0.85 |
| elfamycin | 1 | 0.9 | 0.95 |
| fosfomycin | 1 | 0.96 | 0.98 |
| fusidic_acid | 0.4 | 1 | 0.57 |
| glycopeptide | 1 | 0.99 | 0.99 |
| macrolide-lincosamide-streptogramin | 0.96 | 0.93 | 0.95 |
| multidrug | 0.7 | 0.6 | 0.65 |
| peptide | 0.62 | 0.28 | 0.38 |
| quinolone | 0.75 | 0.96 | 0.84 |
| rifampin | 0.89 | 1 | 0.94 |
| sulfonamide | 1 | 0.93 | 0.96 |
| tetracycline | 0.93 | 0.94 | 0.93 |
| thiostrepton | 1 | 1 | 1 |
| avg/total | 0.94 | 0.93 | 0.93 |

**Table S4**: Prediction results of the genes from MEGARes using the deepARG-LS model trained with our database.
